# Cu–Ferrocene‐Functionalized CaO_2_ Nanoparticles to Enable Tumor‐Specific Synergistic Therapy with GSH Depletion and Calcium Overload

**DOI:** 10.1002/advs.202100241

**Published:** 2021-05-24

**Authors:** Hanjing Kong, Qiang Chu, Chao Fang, Guodong Cao, Gaorong Han, Xiang Li

**Affiliations:** ^1^ State Key Laboratory of Silicon Materials School of Materials Science and Engineering Zhejiang University Hangzhou 310027 P. R. China; ^2^ Department of Surgery Second Affiliated Hospital Zhejiang University School of Medicine Hangzhou 310009 P. R. China; ^3^ ZJU‐Hangzhou Global Scientific and Technological Innovation Center Zhejiang University Hangzhou 311200 P. R. China

**Keywords:** calcium overload, CaO_2_, Cu–ferrocene, GSH depletion, synergistic tumor therapy

## Abstract

The conversion of endogenous H_2_O_2_ into toxic hydroxyl radical (^•^OH) via catalytic nanoparticles is explored for tumor therapy and received considerable success. The intrinsic characteristics of microenvironment in tumor cells, such as limited H_2_O_2_ and overexpressed glutathione (GSH), hinder the intracellular ^•^OH accumulation and thus weaken therapeutic efficacy considerably. In this study, fine CaO_2_ nanoparticles with Cu–ferrocene molecules at the surface (CaO_2_/Cu–ferrocene) are successfully designed and synthesized. Under an acidic condition, the particles release Ca^2+^ ions and H_2_O_2_ in a rapid fashion, while they can remain stable in neutral. In addition, agitated production of ^•^OH occurs following the Fenton reaction of H_2_O_2_ and ferrocene molecules, and GSH is consumed by Cu^2+^ ions to avoid the potential ^•^OH consumption. More interestingly, in addition to the exogenous Ca^2+^ released by the particles, the enhanced ^•^OH production facilitates intracellular calcium accumulation by regulating Ca^2+^ channels and pumps of tumor cells. It turns out that promoted ^•^OH induction and intracellular calcium overload enable significant in vitro and in vivo antitumor phenomena.

## Graphical abstract

For the first time, fine CaO_2_ nanoparticles functionalized with Cu–ferrocene molecules, named as CaO_2_/Cu–ferrocene, are successfully constructed, enabling unique characteristics including hydroxyl radical (^•^OH) induction with self‐supplied H_2_O_2_, glutathione (GSH) depletion, and calcium overload within tumor cells. Considerable in vitro and in vivo anticancer properties are achieved for tumor‐specific therapy.

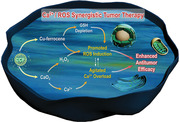

## 1 Introduction

Featured with short‐lived, highly reactive, and oxygen‐containing, reactive oxygen species (ROS) encompass a family of molecules like ^•^O_2_^–^, H_2_O_2_, ^1^O_2_, and hydroxyl radical (^•^OH).^[^ ^1^ ^]^ In general, oxidative stress homeostasis in tumor cells is destroyed by excessive intracellular ROS accumulation, leading to eventual apoptosis.^[^ ^2^ ^]^ A variety of oncology treatments with nanomedicine, based on this ROS‐related phenomenon, have been spawned in the past decade, including photodynamic therapy (PDT),^[^ ^3^ ^]^ sonodynamic therapy (SDT),^[^ ^4^ ^]^ electrodynamic therapy (EDT),^[^ ^5^ ^]^ and chemodynamic therapy (CDT).^[^ ^6^ ^]^ Distinctively, CDT utilizes the intrinsic characteristics of tumor microenvironment (TME) and inhibits tumor progression by the conversion of endogenous H_2_O_2_ into ^•^OH, which is a type of ROS with highest reactivity to cause cellular damage.^[^ ^7^ ^]^ As a typical TME‐responsive therapeutic strategy, CDT presents unique selectivity and specificity for tumor inhibition. Iron‐based compounds are most commonly used as the CDT agent.^[^ ^8^ ^]^ However, ferrous ions are readily oxidized to ferric ions, which are of remarkably weakened catalytic activity.^[^ ^9^ ^]^ In contrast, ferrocene molecules can present high Fenton reaction activity owing to its strong capability in incorporating abundant Fe^2+^ ions, and more importantly its derivatives show considerable potential for cancer therapy by bridging diverse metal ions to form functional nanoparticles.^[^ ^10^ ^]^

Glutathione (GSH), which is overexpressed in the TME,^[^ ^11^ ^]^ is a known antioxidant to scavenge ROS. To promote the magnitude of oxidative stress, GSH consumption has been explored as an effective protocol to synergistically enhance ROS‐based tumor treatments, such as PDT^[^ ^12^ ^]^ and CDT.^[^ ^13^ ^]^ To improve intracellular ROS accumulation, a variety of nanoplatforms have been investigated to consume GSH following the redox reaction with variable valence metal ions in tumor cells, including manganese/copper‐based nanomedicine and so on.^[^ ^14^ ^]^ In addition, the intracellular content of H_2_O_2_ has been recognized as another limitation for maintaining efficacious and lasting CDT, although many types of tumor cells are known to present higher H_2_O_2_ level than the normal.^[^ ^15^ ^]^ It is therefore clear that the implement of sustained H_2_O_2_ supply and GSH suppression with CDT agents can be a highly effective approach to promote ROS induction.

Meanwhile, recent progress indicates that calcium ions directly participate in the regulation of ROS production, including regulating the expression of certain enzymes involved in the process.^[^ ^16^ ^]^ In turn, intracellular calcium homeostasis is achieved through ion channels and pumps on cell membrane.^[^ ^17^ ^]^ Studies have also demonstrated that the intracellular ROS with varied magnitude can influence the activity of related proteins in these channels and pumps. Owing to the strong oxidation of ROS, the cysteine residues of proteins are modified, and thus their conformation and activity vary accordingly.^[^ ^18^ ^]^ For example, the entry of Ca^2+^ ions can be enhanced directly or indirectly by high level of oxidative stress through transient receptor potential (TRP) channels located on the cell membrane. ROS may activate TRP channels directly through Cys modification, such as TRPA1.^[^ ^19^ ^]^ In contrast, as the Ca^2+^ ions efflux pump, the activity of plasma membrane Ca^2+^ ion–ATPase (PMCA) is restrained by high oxidative stress, such as PMCA4.^[^ ^20^ ^]^ Therefore, high level of intracellular ROS favors the accumulation of cytosolic Ca^2+^ ions. Although the underlying relationship between Ca^2+^ ions and cell death is not fully understood, it is clear that excessive presence of Ca^2+^ ions in cytosol can induce cell death, which may be potentially related to disruption of Ca^2+^ ions’ signaling.^[^ ^21^ ^]^ A “milestone” development indicates that the combined induction of intracellular H_2_O_2_ with Ca^2+^ by calcium peroxide nanoparticles can effectively induce apoptosis of tumor cells, which is highly inspiring in developing “green” approaches for tumor therapy.^[^ ^22^ ^]^ It is noteworthy that, in addition to the induction of exogenous Ca^2+^ ions, CaO_2_ can also serve as the source of sustained H_2_O_2_ supply, which is highly demanded in maintaining ^•^OH generation of CDT.^[^ ^23^ ^]^

**Figure 1:**
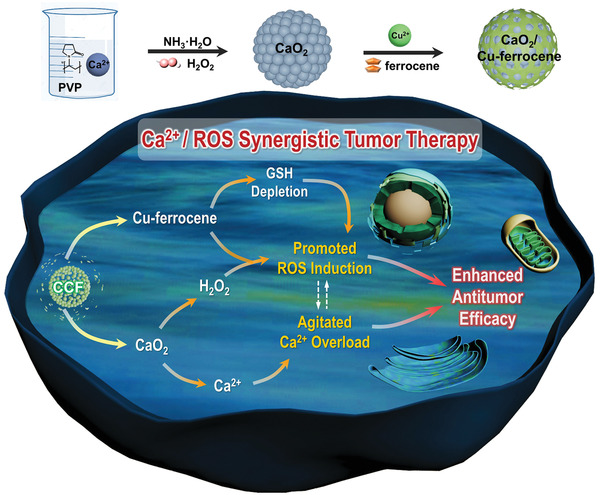
Schematic illustration of the synthesis and functioning procedures of CaO_2_/Cu–ferrocene.

Therefore, herein this study, fine CaO_2_ nanoparticles that are functionalized with Cu–ferrocene molecules at the surface (CaO_2_/Cu–ferrocene, CCF) are designed and successfully synthesized. Owing to the surface protection, CCF can remain at a stable state under a neutral solution. As illustrated in **Figure** **1**, CCF particles can release Ca^2+^ ions and H_2_O_2_ in an acidic intracellular environment. The intracellular H_2_O_2_ is catalyzed by ferrocene molecules to generate ^•^OH following the Fenton reaction. Meanwhile, Cu^2+^ ions enable excellent GSH depletion to enhance the accumulation of ^•^OH. More importantly, the enhanced ^•^OH generation facilitates intracellular calcium accumulation by regulating Ca^2+^ channels and pumps of tumor cells. In consequence, the combined effects of promoted ^•^OH induction and intracellular calcium overload enable significant in vitro and in vivo antitumor efficacy. This multifunctional nanoplatform has successfully implemented current CDT with self‐supplied H_2_O_2_, GSH depletion, and calcium overload, which paves the way to the follow‐on explorations in the intelligent systems for tumor‐specific therapy.

## 2 Results and Discussion

**Figure 2:**
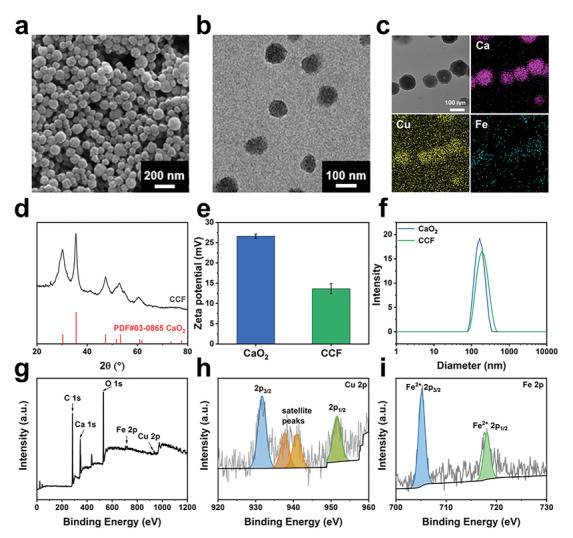
Characteristics of CaO_2_/Cu–ferrocene. a) Scanning electron microscopy (SEM), b) transmission electron microscopy (TEM), c) element mapping images, and d) XRD pattern of CaO_2_/Cu–ferrocene. e) Zeta potential and f) hydrodynamic dimension of CaO_2_ and CaO_2_/Cu–ferrocene particles. g) XPS spectrum of CaO_2_/Cu–ferrocene. h) XPS high‐resolution spectrum of Cu 2p. i) XPS high‐resolution of Fe 2p.

CCF nanoparticles were synthesized following a two‐step approach, as demonstrated in Figure 1. CaO_2_ nanoparticles, prepared via a wet‐chemical process reported,^[^ ^24^ ^]^ present a spherical morphology with uniform size distribution (**Figure** **2**a). When Cu–ferrocene molecules are grafted at the surface of CaO_2_, the nanoparticles show an improved dispersion with a mean diameter of ≈80 nm (Figure 2b). No clear variation in the particle morphology can be observed from transmission electron microscopy (TEM) images (Figure S1a,b, Supporting Information). The elements of Cu and Fe distribute across the CCF particles in a uniform manner (Figure 2c). Comparing to the as‐prepared CaO_2_ nanoparticles, the X‐ray diffraction (XRD) spectrum of CCF presents the consistent characteristics with three dominant peaks at 30.1°, 35.6°, and 47.3°, which are attributed to pure CaO_2_ phase (PDF#03‐0865; Figure 2d; Figure S1c, Supporting Information), and no additional impurities are observed. The findings indicate that the surface coverage of Cu–ferrocene is amorphous. The surface potentials of CaO_2_ and CCF are ≈26.6 and ≈13.6 mV, respectively (Figure 2e). The decreased surface potential is due to the ferrocene molecules with negative charge, which are grafted at the particles surface. As shown in Figure 2f, compared with CaO_2_, the hydrodynamic size of CCF increases from ≈164 to ≈190 nm, as expected. The characteristic peak at ≈258 nm presents in UV–vis absorbance spectrum of CCF (Figure S2, Supporting Information). The characteristic peaks of IR spectra located at ≈798.5 and ≈1562.2 cm^–1^ verify that Cu–ferrocene is successfully grafted in CCF (Figure S3, Supporting Information). The C=O stretching peak is shifted to lower wavenumbers by a marginal magnitude due to the weak chemical coordination of poly(vinyl pyrrolidone) (PVP) and copper.^[^ ^25^ ^]^ The X‐ray photoelectron spectroscopy (XPS) spectrum of CCF indicates the elements of Ca, Cu, Fe, and O (Figure 2g). The valence state of copper ions is bivalent according to the Cu 2p XPS spectrum with Cu^2+^ ions peaks at 932 and 952 eV accompanied by satellite peak (Figure 2h). The spectrum of Fe 2p indicates that the iron ions in CCF are mainly composed of Fe^2+^ ions (Figure 2i). Two O 1s peaks at 530 and 528.5 eV correspond to O—O and C=O, respectively, indicating the co‐existence of CaO_2_ and PVP molecules (Figure S4, Supporting Information).

**Figure 3:**
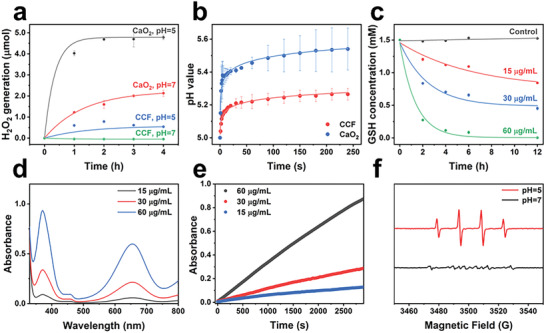
a) Release profiles of H_2_O_2_ from CaO_2_ and CaO_2_/Cu–ferrocene under different pH. b) pH variation in acetate buffer solution with the addition of CaO_2_ and CaO_2_/Cu–ferrocene. c) GSH consumption of CaO_2_/Cu–ferrocene with different concentrations. d) UV–vis absorbance spectra. e) Time‐course absorbance at 655 nm with different concentrations of CaO_2_/Cu–ferrocene in TMB solution. f) ESR spectra of CaO_2_/Cu–ferrocene at different pH values.

When immersed in the deionized water, bare CaO_2_ particles hydrolyze to H_2_O_2_ or O_2_, and lower pH induces more rapid generation of H_2_O_2_, as expected (**Figure** **3**a; Figure S5, Supporting Information). In contrast, after being grafted with Cu–ferrocene, the release kinetics of H_2_O_2_ from CCF is significantly postponed. In addition to the protection effect of Cu–ferrocene, this phenomenon is also attributed to the effective consumption of H_2_O_2_ by the Fe^2+^ ions of ferrocene via Fenton reaction. Under a condition with lower pH, CCF could effectively release H_2_O_2_, implying its responsive properties to the acidic tumor microenvironment. Meanwhile, CCF hardly induces H_2_O_2_ under a neutral condition, verifying that the particles present excellent chemical stability in the neutral physiological environment. In addition, when the hydrolysis of CaO_2_ occurs, a large amount of Ca(OH)_2_ is produced, rising the pH values accordingly. Compared with CaO_2_, the pH variation caused by CCF is significantly weakened, verifying the protection effect of Cu–ferrocene in CCF (Figure 3b). To further confirm the improved stability of CCF under neutral conditions, CCF is dispersed and immersed in phosphate‐buffered solution (pH = 7.4). The observation using TEM indicates that CCF presents superior stability comparing to CaO_2_ (Figure S6, Supporting Information). It is known that GSH acts as an ROS scavenger in tumor cells, and thus overexpressed GSH usually weakens the efficacy of ROS‐based treatment. To explore its GSH depletion effect, CCF with varied concentrations is added in the GSH solution and examined at different time intervals (0, 2, 4, 6, and 12 h). It is clear that, comparing to the blank control, the addition of CCF induces sustained reduction of GSH concentration (Figure 3c; Figures S7 and S8, Supporting Information). Higher CCF concentration enables more rapid decline in the GSH concentration. After eliminating the interference of CaO_2_, Cu–ferrocene alone can also lead to the decrease of GSH content, confirming that Cu^2+^ ions in CCF could effectively consume GSH via a reduction reaction (Figure S9, Supporting Information).

As shown in the results of 3,3′,5,5′‐tetramethylbenzidine (TMB) assay, CCF can effectively generate ^•^OH under an acidic condition, and higher concentration induces more agitated ^•^OH generation according to the raising absorbance peaks (Figure 3d). CCF with increased concentration accelerates the generation of ^•^OH, as verified in Figure 3e. To uncover the mechanism, UV–vis absorbance spectra of TMB solution with the addition of ferrocene and ferrocene/H_2_O_2_ were compared. In the presence of H_2_O_2_, the characteristic absorbance peak of ox‐TMB presents, while no signal for the ^•^OH generation is observed when ferrocene alone is used (Figure S10a, Supporting Information). The findings indicate that ^•^OH is generated from the Fenton reaction between ferrocene molecules and H_2_O_2_. Although CaO_2_ may attenuate the acidity of the solution, the Fenton reaction triggered by ferrocene molecules can still effectively occur (Figure S10b, Supporting Information). The examination using electron spin‐resonance (ESR) spectrometry presents that CCF, in an acidic condition, shows a typical spectrum with fourfold peaks with the intensity ratio of 1:2:2:1, which is attributed to ^•^OH (Figure 3f). In comparison, no clear signals can be observed to CCF under a neutral condition. The findings illustrate that CCF synthesized can act as a Fenton catalyst to effectively induce ^•^OH with self‐supplied H_2_O_2_ under an acidic condition.

**Figure 4:**
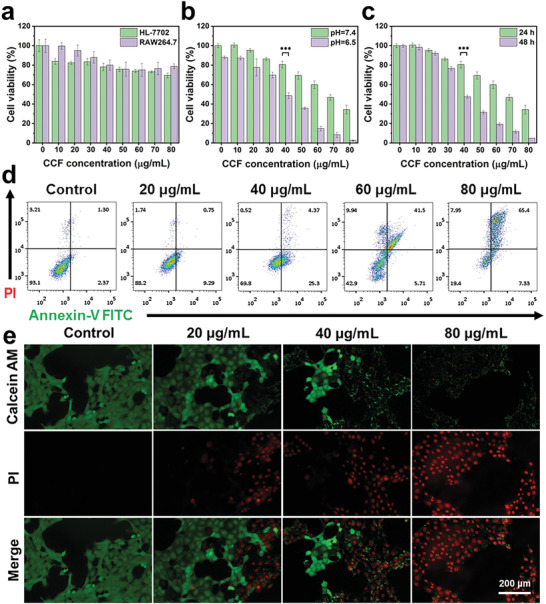
a) HL‐7702 and RAW 264.7 cell viabilities with different concentrations of CaO_2_/Cu–ferrocene. 4T1 viabilities with different concentrations of CaO_2_/Cu–ferrocene at varied b) pH and c) incubation time. d) Flow cytometry analysis and e) Live and Dead cell staining of 4T1 cells with different concentrations of CaO_2_/Cu–ferrocene (***p < 0.001, **p < 0.01, or *p < 0.05).

The antitumor properties of CCF were initially examined at a cellular level. To verify the toxicity to normal cells, CCF with varied concentrations was incubated with HL‐7702 (human liver cell) and RAW264.7 (mouse mononuclear macrophages) lines, and no clear negative effect to the cell viability was observed (**Figure** **4**a). In contrast, the viabilities of 4T1 tumor cells were highly dependent on the concentration of CCF (Figure 4b). At the same concentration, the cytotoxicity of CCF to tumor cells was remarkably enhanced under an acidic condition. This is attributed to the pH‐dependent Fenton reaction of CCF. In addition, the cytotoxic effect of CCF was related to the incubation time, as expected (Figure 4c). The flow cytometry assay was used to evaluate the apoptosis of 4T1 cells in a more quantitative manner. The apoptosis rates of cell groups treated with blank control and CCF with different concentrations (20–80 µg mL^−1^) were ≈3.7%, ≈10.0%, ≈29.7%, ≈47.2%, and ≈72.7%, respectively (Figure 4d). The inhibition effect to tumor cells and the suppression of cell proliferation of CCF were further verified by the Live and Dead staining (Figure 4e) and colony efficiency assay (Figure S11, Supporting Information), respectively, showing consistent to the findings above.

**Figure 5:**
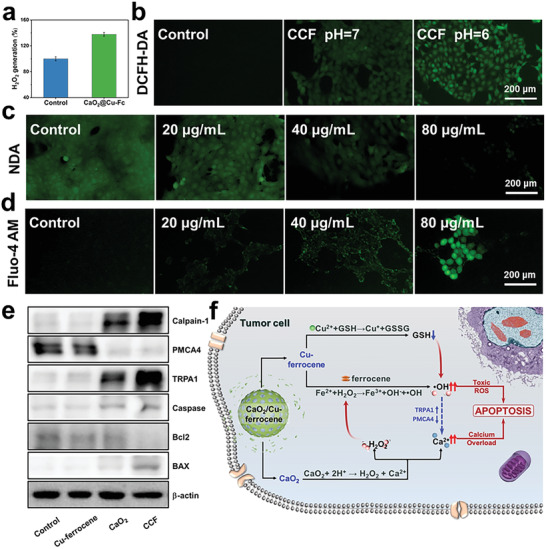
a) Intracellular H_2_O_2_ in 4T1 cells cultured without or with CaO_2_/Cu–ferrocene. b) Fluorescence images of 4T1 cells incubated with CaO_2_/Cu–ferrocene and varied pH after DCFH‐DA staining for ROS detection. Fluorescence images of 4T1 cells incubated with different concentrations of CaO_2_/Cu–ferrocene after staining for c) intracellular GSH depletion and d) intracellular Ca^2+^ ions’ accumulation. e) Expression of Calpain‐1, PMCA4, TRPA1, Caspase‐3, Bcl‐2, and BAX in 4T1 cells treated with different groups in inducing cellular apoptosis. f) Schematic diagram of functioning of CaO_2_/Cu–ferrocene as a multifunctional platform for promoted ROS induction and calcium overload.

The underlying mechanism of in vitro antitumor properties of CCF was revealed. It is known that CaO_2_ induces H_2_O_2_ and Ca^2+^ ions under the acidic condition. As shown in **Figure** **5**a, intracellular H_2_O_2_ content in 4T1 cells increases after the incubation with CCF. According to the intracellular ROS assay using 2′,7′‐dichlorodihydrofluorescein diacetate (DCFH‐DA), CCF induces clear green fluorescence in tumor cells, and the fluorescence intensity is enhanced when the pH of incubation medium is reduced from 7 to 6, indicating that the intracellular ROS is promoted under the acidic condition (Figure 5b). To distinguish ^•^OH from H_2_O_2_, ROS Brite hydroxyphenyl fluorescein (HPF) was used to selectively detect ^•^OH. After the treatment of CCF, clear enhancement of green fluorescence in 4T1 cells indicated that CCF may effectively convert the generated H_2_O_2_ to toxic ^•^OH (Figure S12, Supporting Information). The variation of GSH content within tumor cells cultured with CCF of different concentration is examined. As shown in Figure 5c and in Figures S13 and S14a (Supporting Information), the green fluorescence of intracellular GSH is quenched with the increased CCF concentration. The findings suggest that Cu^2+^ ions released from CCF could effectively consume intracellular GSH, which is highly expressed in tumor cells.

In general, ROS can alter protein conformation and desensitize calcium‐ion channels and pumps followed by uncontrollable accumulation of intracellular Ca^2+^ ions. The intensive green fluorescence and promoted expression of calpain‐1 reflect the increased enrichment of Ca^2+^ ions in tumor cells (Figure 5d,e; Figures S14b and S15, Supporting Information). The known fact is that the intracellular calcium enrichment could effectively hinder the accurate transmission of calcium signals and induce cell death. In this study, the western blot assay indicates that the expression of PMCA4 is suppressed when that of TRPA1 is strengthened, which is potentially induced by the ROS oxidation. PMCA4, which extrudes Ca^2+^ ions from the tumor cell,^[^ ^18^ ^]^ is inactivated by high oxidative stress. Meanwhile, ROS activates TRP channels directly through oxidative modification of cysteine,^[^ ^19^ ^]^ resulting in the enhancement of Ca^2+^ ions’ entry. The inhibited efflux and agitated influx of Ca^2+^ ions lead to intracellular calcium enrichment. Compared with CaO_2_, CCF further enhances the expression of TRPA1, enhancing the influx of Ca^2+^ ions into the cells. The findings indicate that the intensive ^•^OH generation could further agitate the accumulation of intracellular calcium. In all experimental groups, the 4T1 cells treated with CCF present the most significant variation in the expression of apoptotic‐pathway‐related proteins. After treated with CCF, the suppressed Bcl‐2 expression, the promoted levels of BAX and Caspase‐3 in 4T1 indicate that the combination of excessive ROS and calcium overload activates the mitochondrial apoptosis pathway.

Overall, the apoptosis induction for tumor cells by CCF becomes clear. As demonstrated in Figure 5f, after being uptaken by tumor cells, CCF degrades into CaO_2_ and Cu–ferrocene owing to the intracellular acidic environment. CaO_2_ reacts with H^+^ to induce H_2_O_2_ and Ca^2+^. The increased intracellular H_2_O_2_ content combining both endogenous and exogenous H_2_O_2_ enhances the Fenton reaction of Fe^2+^ and thus promotes the generation of ^•^OH. Meanwhile, Cu ions induced by Cu–ferrocene effectively consume GSH, preventing the potential ROS reduction. In addition to the exogenous Ca^2+^ induced by CCF, the promoted intracellular ROS suppresses efflux and agitates influx of Ca^2+^ ions, favoring the calcium enrichment in tumor cells. The combination of promoted ^•^OH and calcium overload results in the significant apoptosis of tumor cells in a selective manner.^[^ ^22^, ^26^ ^]^

**Figure 6:**
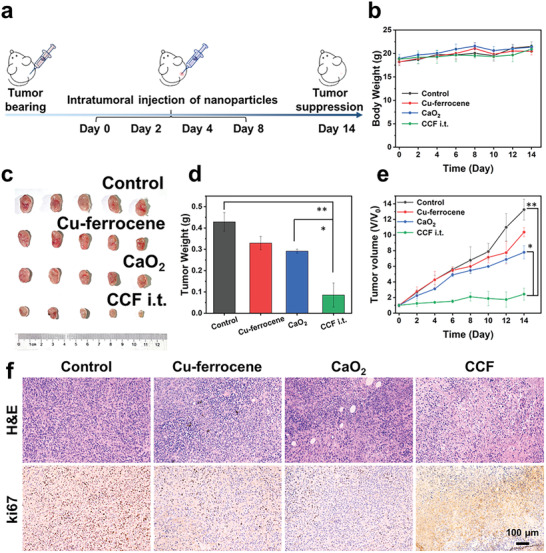
In vivo assay. a) Experimental procedures of injection treatment. b) Variation of body weight over 14 days. c) Tumor photographs and d) tumor weights from different mice groups after 14 days. e) Variation of tumor volume in different groups during 14 day treatment. f) Images of H&E‐ and ki67‐stained tumor slices from different groups after 14 days (***p < 0.001, **p < 0.01, or *p < 0.05).

Following the in vitro study, the in vivo antitumor phenomenon of CCF was evaluated in 4T1 tumor xenografts after intratumoral injection on days 0, 2, 4, and 8, as demonstrated in **Figure** **6**a. Tumor‐bearing mice were stochastically divided into four groups and intratumorally administrated with different treatments, including saline (Control), Cu–ferrocene, CaO_2_, and CCF. All mice did not show apparent variations in body weight (Figure 6b), indicating that no significant side effect was induced by the injection of Cu–ferrocene, CaO_2_, and CCF. Comparing to the control group, Cu–ferrocene complex presents a certain inhibitory effect on tumor due to the ROS induced in tumor microenvironment containing H_2_O_2_. Meanwhile, the injection of CaO_2_ induced inhibition to the tumor growth by a marginal promoted magnitude, which is attributed to Ca^2+^ accumulation at the tumor site. In contrast, with the combination of Cu–ferrocene, CCF presented the most considerable tumor inhibition (Figure 6c), which is also verified by the variation of tumor weight (Figure 6d) and volume (Figure 6e). After the administration of different treatments, morphological features of cell apoptosis were examined by hematoxylin and eosin (H&E) and ki67 staining (Figure 6f). It is clear that the injection of CCF induced significant vacuolation, nuclear shrinkage, and cell membrane rupture, which displayed the strongest tumor apoptosis. The ki67 positive cells were strikingly decreased compared with all other groups, indicating the most significant suppression in tumor proliferation enabled by CCF. The findings verify the considerable in vivo antitumor properties of CCF.

**Figure 7:**
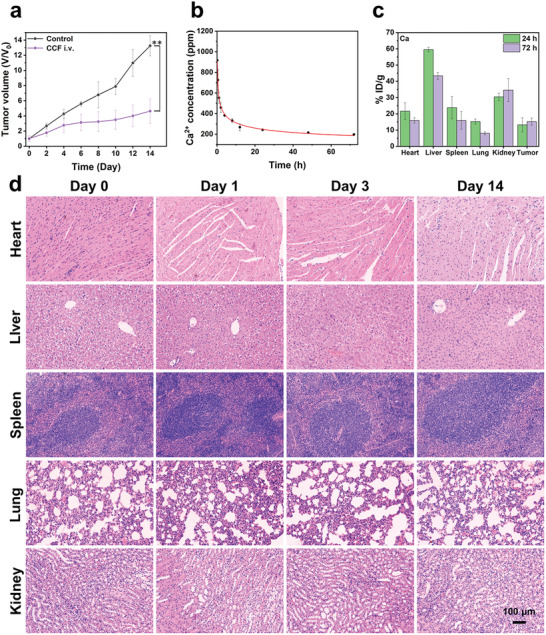
a) Variation of tumor volume in Group 5 treated with intravenous injection of CCF. b) In vivo pharmacokinetic curves in 72 h. c) Distribution of nanoparticles in different organs at 24 and 72 h after injecting with CCF. d) H&E‐stained slices from different organs on days 0, 1, 3, and 14 (***p < 0.001, **p < 0.01, or *p < 0.05).

To demonstrate the potential in the possible clinical translation, CCF nanoparticles were injected via tail intravenous (i.v.) on days 0, 4, and 8, respectively. As shown in Figure S17a (Supporting Information), stable body weight showed no clear discrepancy within 14 days. Compared with the control group, tumors were restrained to a large extent in the group of CCF, demonstrating the selective tumor suppressive effect (**Figure** **7**a; Figure S17a–d, Supporting Information).

In addition, the in vivo pharmacokinetics, biodistribution, and biosafety of CCF were examined. The in vivo pharmacokinetic curves indicated that CCF could be maintained in blood circulation for a certain time of period (Figure 7b), favoring the enrichment of CCF at the tumor site (Figure 7c; Figure S18, Supporting Information). After 72 h, the content of elements in organs decreased, while that at the tumor site slightly increased, suggesting that nanoparticles can accumulate at the tumor site while being metabolized in normal organs. There was no clear difference in the number of red blood cells and white blood cells during the treatment (Figure S19a, Supporting Information). Meanwhile, the decreased contents of alanine transaminase (ALT) and blood urea nitrogen (BUN) indicated that the damage to the liver and kidney was alleviated (Figure S19b,c, Supporting Information). Histopathological H&E staining of main organs suggested the negligible side effects of CCF on mice (Figure 7d). After 30 days, the mice from both groups were dissected, and a clear pulmonary metastasis was observed in the control group, while mice after treatment via the i.v. injection of CCF did not present the clear sign for the pulmonary metastasis (Figure S20, Supporting Information). The H&E‐stained images of lung slices verified the findings above, implying that the CCF investigated in this study appears to be a highly potential nanoplatform to tumor treatment.

## 3 Conclusions

In this study, fine CaO_2_ nanoparticles that are functionalized with Cu–ferrocene molecules at the surface (CCF) are successfully designed and synthesized. Under an acidic condition with tumor cells, CCF releases Ca^2+^ ions and H_2_O_2_ in a rapid fashion, while the particles remain stable in the solution with neutral pH owing to the surface protection by Cu–ferrocene. In addition, agitated production of ^•^OH occurs following the Fenton reaction of H_2_O_2_ and ferrocene molecules, and GSH is consumed by Cu^2+^ ions to avoid the potential ^•^OH scavenge. More importantly, in addition to the exogenous Ca^2+^ ions induced by CaO_2_, the enhanced ^•^OH generation facilitates intracellular calcium accumulation by regulating Ca^2+^ channels and pumps of tumor cells. In consequence, promoted ^•^OH induction and agitated calcium overload in tumor cells achieved by CCF enable significant in vitro and in vivo antitumor phenomena. This multifunctional nanoplatform has successfully implemented unique characteristics, including ^•^OH induction with self‐supplied H_2_O_2_, GSH depletion, and calcium overload, which is anticipated to inspire a series of follow‐on explorations in the intelligent systems for tumor‐specific therapy.

## Conflict of Interest

The authors declare no conflict of interest.

## Supporting information

Supporting Information

## Data Availability

The data that support the findings of this study are available from the corresponding author upon reasonable request.
