# Supplementary material for: Cu–Ferrocene‐Functionalized CaO2 Nanoparticles to Enable Tumor‐Specific Synergistic Therapy with GSH Depletion and Calcium Overload
Source: Adv Sci (Weinh). 2021 May 24;8(14):2100241. doi: 10.1002/advs.202100241 (PMC8292872; doi:10.1002/advs.202100241)
Supplement: Supplementary file 1 — Supporting Information [file ADVS-8-2100241-s001.pdf]

WILEY-VCH

© 2021 Wiley-VCH GmbH

ADVANCED SCIENCE
Open Access

Supporting Information

for *Adv. Sci.*, DOI: 10.1002/advs.202100241

Cu-ferrocene Functionalized CaO_2 Nanoparticles to enable Tumor-Specific Synergistic Therapy with GSH Depletion and Calcium Overload

*Hanjing Kong Qiang Chu, Chao Fang, Guodong Cao, Gaorong Han and Xiang Li, **

Supporting Information

Cu-ferrocene Functionalized CaO_2 Nanoparticles to enable Tumor-Specific Synergistic Therapy with GSH Depletion and Calcium Overload

Hanjing Kong, [1,#] *Qiang Chu,* [1,#] *Chao Fang,* [1] *Guodong Cao,* [2] *Gaorong Han* [1] *and Xiang Li,* [1,3]*

[1] State Key Laboratory of Silicon Materials, School of Materials Science and Engineering, Zhejiang University, Hangzhou 310027, P.R. China

[2] Department of Surgery Second Affiliated Hospital, Zhejiang University School of Medicine, Hangzhou 310009, P. R. China

[3] ZJU-Hangzhou Global Scientific and Technological Innovation Center, Zhejiang University, Hangzhou, 311200, P.R. China.
[#] Authors with equal contribution
* Corresponding author: xiang.li@zju.edu.cn (XL)

Keywords: Cu-ferrocene; CaO_2; GSH depletion; calcium overload; synergistic tumor therapy.

Experimental Section

Chemical and reagents. Calcium chloride anhydrous ($CaCl_2$, AR), hydrogen peroxide (H_2O_2, 30 wt%), ammonia solution (NH_3•H_2O, 25wt%), copper (II) acetate monohydrate (($CH_3COO)_2Cu$•H_2O, AR), N,N-dimethylformamide (DMF, AR) and ethanol anhydrous were purchased from Sinopharm Chemical Reagent. 1,1'-ferrocenedicarboxylic acid ($Fc(COOH)_2$, >98%) was purchased from Aladdin-Reagent. 3,3',5,5'-Tetramethylbenzidine (TMB), 5,5-Dimethyl-1-pyrroline N-oxide (DMPO), dichlorofluorescein diacetate (DCFH-DA) were obtained from Sigma-Aldrich. Horseradish peroxidase (HRP) was purchased from Hefei Bomei Biotechnology. Polyvinyl pyrrolidone (PVP, 0.99), Fluo-4 AM, naphthalene-2,3-dicarboxaldehyde (NDA) and Cell Count Kit-8 (CCK-8) were purchased from Meilunbio. Calcein AM and propidium iodide (PI) probes were obtained from Dojindo (Shanghai, China). Anti-Calpain 1 antibody, Anti-Calcium Pump PMCA4 ATPase antibody, Anti-TRPA1/TSA antibody were purchased from Abcam. HRP-labeled goat anti-rabbit IgG (H+L) and ECL western blotting system were obtained from Beyotime Biotechnology.

Characterization. Microstructure images of samples were obtained using transmission electron microscopy (TEM, HITACHI HT-7700) and field-emission scanning electron microscopy (FESEM, Phenom Pharos), respectively. Zeta potential and dynamic light scattering were measured by Zetasizer Nano-ZS (0.3-10000 nm). X-ray diffraction pattern was obtained by X-ray diffractometer with Cu Kα radiation (XRD, X'pert PRO MPD). The chemical composition and valence states of elements were measured by X-ray photoelectron spectroscopy (XPS, AXIS Supra). The UV-visible absorbance was examined by UV-vis spectrophotometer (Shimadzu, UV-2600). Hydroxyl radicals was detected by electron spin resonance spectroscopy (EPR, Bruker A300). Cell Counting Kit-8 (CCK-8), H_2O_2 and GSH contents were recorded using a microplate reader. The fluorescence images of Live and Dead, intracellular reactive oxygen species (ROS) and Ca^{2+} ions were obtained by an inverted fluorescent microscope (Nexcope, The USA).

Synthesis of CaO_2 and CaO_2/Cu-ferrocene (CCF) nanoparticles. CaO_2 spherical aggregates were synthesized following protocol the procedures reported previously [1]. $CaCl_2$ (0.1 g) and PVP (0.35 g) were dissolved in 15 mL ethanol, and 1 mL NH_3•H_2O (0.8 M) was added in sequence under stirring. Subsequently, 0.2 mL H_2O_2 (1 M) was added dropwise to the solution using a syringe pump. Finally, the milky white solution was centrifuged at 12000 rpm for 10 min and washed three times with ethanol. The white precipitant was redispersed in 5 mL ethanol for further use.

CCF was prepared *via* in situ assembly. In brief, 5 mg CaO_2 was dispersed into 2 mL DMF, and 16 mg PVP was dissolved into 16 mL DMF under stirring. Two solutions were then mixed. Subsequently, 0.2 mL $(CH_3COO)_2Cu$•H_2O (0.1 M) and 0.2 mL $Fc(COOH)_2$ (0.1 M) were added into the solution. After stirring at 80 °C for 1 h, the product was obtained by centrifugation (10000 rpm, 10 min) and washed twice with DMF and ethanol, respectively.

Functional characteristics of CCF.

The elemental composition of CaO_2 and CCF. The content of Ca, Fe and Cu elements in CCF (or Ca element in CaO_2) were measured by inductively coupled plasma-mass spectrometry (ICP-MS) and calculated as Ca, Fe and Cu percentage in CaO_2 or CCF.

H_2O_2 generation. In a typical process, 1 mg CCF (or CaO_2 containing the same amount of Ca^{2+} ions) was dispersed in 1 mL acetate buffer solution (ABS) at varied pH values. At different time intervals, the supernatant was collected by centrifugation for examinations. 300 μL HRP (1 U/mL) and 20 μL supernatant were added in 1.68 mL phosphate buffer solution (PBS, pH = 5.8). After 10 minutes' reaction, 300 μL TMB (2 mM) was added. The total amount of H_2O_2 generated from CaO_2 and CCF was quantitatively determined according to the UV-vis absorbance standard curve of ox-TMB at 372 nm

pH variation. 1 mg CCF (or CaO_2 containing the same amount of Ca^{2+} ions) was dispersed into 1 mL ABS (initial pH = 5). pH values were recorded with a real-time pH meter.

Stability of CCF and CaO_2 nanoparticles. CCF (or CaO_2 containing the same amount of Ca^{2+} ions) was dispersed into PBS (pH=7.4) and incubated for certain time intervals (1 h and 4 h). Subsequently, the morphology of nanoparticles was observed by TEM.

GSH depletion. Different quantities of CCF were dispersed in 6 mL GSH solution (1.5 mM, pH=7) at 37 °C. 0.5 mL supernatant was collected from the solution after centrifugation at different time points of the reaction (0, 2, 4, 6 and 12 h). Subsequently, 2.5 mL PBS and 50 μL 5,5'-Dithiobis-(2-nitrobenzoic acid) (10 mM, DTNB was used as the indicator of GSH) were added into the supernatant. The total amount of GSH was determined quantitatively according to the UV-vis absorbance standard curve of GSH at 407 nm.

To exclude the consumption of GSH concentration by the hydrogen peroxide induced by the hydrolysis of CaO_2, the examination was carried out using CaO_2 and Cu-ferrocene, respectively. The reaction time was set at 1 hour, and the experimental procedure was maintained the same as stated above.

•OH generation. •OH generation was detected by the chromogenic reaction of TMB. Briefly, TMB (8 mM) was added into 3 mL ABS (0.1 M, pH = 5) with CCF at different concentrations. The absorption spectra and kinetic curves were recorded by a UV-vis spectrometer. The generation of •OH was further identified by electron spin resonance (ESR) spectroscopy with 5, 5-Dimethyl-1-pyrroline N-oxide (DMPO) as a spin trap, which can be recognized with a characteristic 1:2:2:1 hydroxyl radical signal.

In vitro study.

Cell viability assay. The cell viability of CCF was examined by CCK-8. Cell compatibility was evaluated by normal cell lines, HL-7702 (human liver cell) and RAW264.7 (mouse mononuclear macrophages). Briefly, cells (10,000 per well) were seeded into a 96-well plate for 12 h. The medium was removed, and fresh medium containing CCF (0-80 μg/mL) was added and incubated for further 24 h. 10 μL CCK-8 was added per well and incubated for 1 h. The results were acquired using a microplate reader for absorbance at 450 nm.

In addition, the *in vitro* antitumor properties were examined by CCK-8 assay using 4T1 cells (mouse breast cancer cell). Briefly, 4T1 cells (10,000 per well) were seeded into a 96-well plate for 12 h. Then, the medium was removed and fresh medium containing CCF (0-80 μg/mL) was added for further 24 h. The results were acquired using a microplate reader at 450 nm. To simulate acidic tumor microenvironment, the pH of cell culture was set at 6.5 by the addition of HCl solution (1 M, 15 μL) into the medium (980 μL), and CCF was added subsequently.

The Live and Dead assay was also used to confirm the cytotoxicity of CCF to tumor cells. After being seeded in 6-well plates and incubated for 12 h, 4T1 cells were treated with different concentration of CCF. After 12 h, the cells were stained with the calcien AM/propidium iodide (PI) solution for 30 min and observed at 480 nm and 525 nm, respectively.

Flow cytometry assay. 4T1 cells were seeded in 6-well plates and incubated for 12 h. The cells were treated with different concentration of CCF for another 24 h. Cell apoptosis was analyzed by flow cytometer after incubation with Annexin V-TITC/ PI apoptosis detection kit in dark for 15 min.

Colony forming efficiency assay. 4T1 cells were seeded in 6-well plates at a low density and incubated for 12 h. The medium was replaced with fresh 1640 solution containing with different concentrations of CCF. After 10 days, the cells were washed with PBS and fixed with 1 mL 4% formaldehyde solution. Finally, 0.5 mL crystal violet staining solution was added and washed after 30 min.

Intracellular ROS and Ca^{2+} ions examination. The production of intracellular ROS by the particles was detected by dichlorofluorescein diacetate (DCFH-DA) probe. Briefly, 4T1 cells were seeded into 6-well plates and incubated at 37 °C for 12 h. After removal of the medium, the cells were incubated with different concentrations of CCF for 12 h. Subsequently 10 μL DCFH-DA (20 μM) was added into the well. After 30 min of incubation in dark, the medium was discarded and washed with PBS for three times. The results were observed using an inverted fluorescent microscope at 480 nm. To further examine the content of •OH in the ROS, ROS Brite^{TM} HPF (10 μM) was used to detect •OH in a selective manner [2].

Fluo-4 AM is a cell-permeable Ca^{2+} indicator, which reacts with the esterase within the cell to generate non-fluorescent Fluo-4. The presence of Ca^{2+} ions endows Fluo-4 with strong fluorescence. To detect excessive Ca^{2+} ions, Fluo-4 AM (10 μM) was applied in accordance with the same procedures above.

In order to assess the magnitude of calcium overload, the intracellular calcium content was examined by inductively coupled plasma optical emission spectrometer (ICP-OES).

Intracellular H_2O_2 generation. Intracellular H_2O_2 content was examined by a H_2O_2 assay kit. Briefly, 4T1 cells were seeded into 6-well plates and incubated at 37 °C for 12 h. After

removal of the medium, the cells were incubated with different concentrations of CCF. After 8 h, cell lysis buffer was added into the well and the supernatant was collected by centrifugation. H_2O_2 assay kit was then added, and the results were recorded by a microplate reader at 560 nm.

Intracellular GSH consumption. The membrane-permeant naphthalene-2,3-dicarboxaldehyde (NDA), an extremely sensitive fluorescence probe, was used to determine intracellular glutathione content [3]. NDA can react with GSH to produce strong fluorescence isoindole adducts. The specific operation was similar to ROS detection, and the concentration of working solution was adjusted at 50 μM.

The GSH content was quantitatively detected using DTNB chromogenic method. After incubation with 40 μg/mL CCF for 8 h, cell lysis buffer was added into the well, and the supernatant was collected by centrifugation. Then DTNB was added and the results were recorded by a microplate reader at 405 nm.

Western blot. After being treated with different samples, 4T1 cells were lysed by RIPA lysis buffer with 1% phenylmethanesulfonyl fluoride (PMSF), and the content of total protein were quantified by BCA assay kit. 50 μg protein was loaded onto an SDS-PAGE, transferred to the PVDF membranes and then blocked by 5% skim milk for 2 h. Subsequently, the PVDF membranes were incubated with primary antibodies for 12 h at 4 °C and secondary antibodies for 2 h at room temperature. After washing with TBST (Tris-buffered saline containing 0.1% Tween 20 buffer) for 5 times, the PVDF membranes were visualized by the enhanced ECL assay kit.

In vivo study. All animal experiments were performed in accordance with the guidelines of the animal ethics committee of the Biological Resource Centre of the Agency for Science, Technology and Research, Zhejiang University. Four-week old female Balb/c mice were purchased from Zhejiang Academy of Medical Science. The mice were randomly divided into

five groups (n = 5). To establish the xenograft model, 4T1 cells ($1x10^6$ cells per mouse) were injected subcutaneously into the right side back of mouse. The tumor volume was calculated from the following equation

$$V = \frac{length \times width^2}{2}$$

Seven days after inoculation, 4T1 tumor-bearing mice were intratumorally injected at day 0, 2, 4 and 8 with different formulations ([CCF] = 15 mg/kg): (1) saline (control group); (2) Cu-ferrocene solution; (3) CaO_2; (4) CCF. In addition, mice in group 5 were intravenously injected at day 0, 4 and 8 with CCF (37.5 mg/kg). The tumor volume and body weight were measured every two days. After 14 days' treatment, tumors and main organs were collected from the sacrificed mice for H&E and ki67 staining. Blood sample was collected for blood routine and serum biochemical analysis.

In vivo blood circulation and bio-distribution. 4T1 tumor-bearing mice were intravenously injected with CCF (37.5 mg/kg). At pre-set time points (10 min, 30 min, 1 h, 2 h, 4 h, 8 h, 12 h, 24 h, 48 h and 72 h), 30 μL blood was collected from each mouse. The mice were sacrificed at day 1, 2 and 3 after intravenous injection for the examination of Ca and Fe content in liver, spleen, kidney, heart, lung, tumor as well as blood sample by ICP-OES.

Statistical analysis. All data in this article are presented as mean ± standard deviations. Student's t-tests were used to evaluate the comparison results between experimental groups. Variations in the data were considered to be significant when $***p < 0.001$, $**p < 0.01$ or $*p < 0.05$.

Reference

[1] S. Shen, M. Mamat, S. Zhang, J. Cao, Z. D. Hood, L. Figueroa-Cosme, Y. Xia, *Small* **2019**, 15, 1902118.

[2] B. R. You, S. Z. Kim, S. H. Kim, W. H. Park, *Mol Cell Biochem* **2011**, 357, 295.

[3] Q. Chu, M. Chen, D. Song, X. Li, Y. Yang, Z. Zheng, Y. Li, Y. Liu, L. Yu, Z. Hua, X. Zheng, *Int. J. Biol. Macromol.* **2019**, 123, 1115.

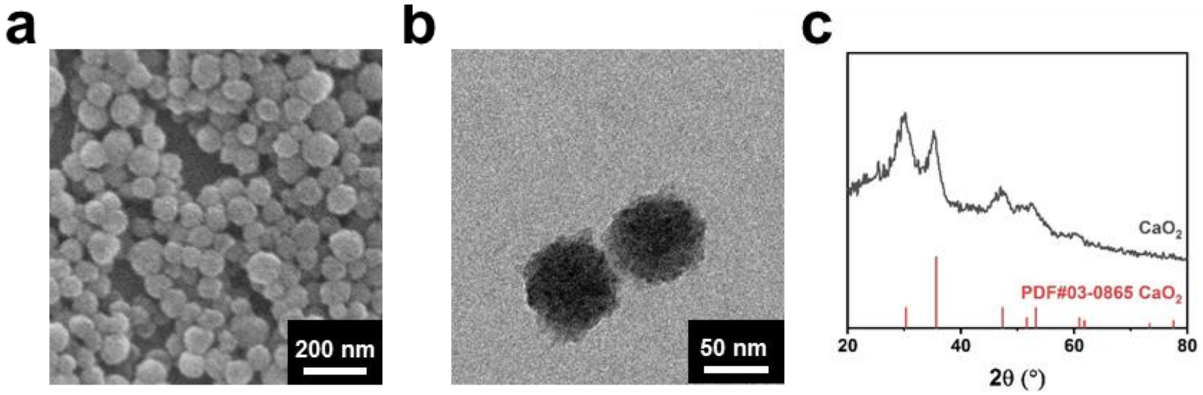

Figure S1. (a) SEM image, (b) TEM image and (c) XRD pattern of as-prepared CaO_2 nanoparticles.

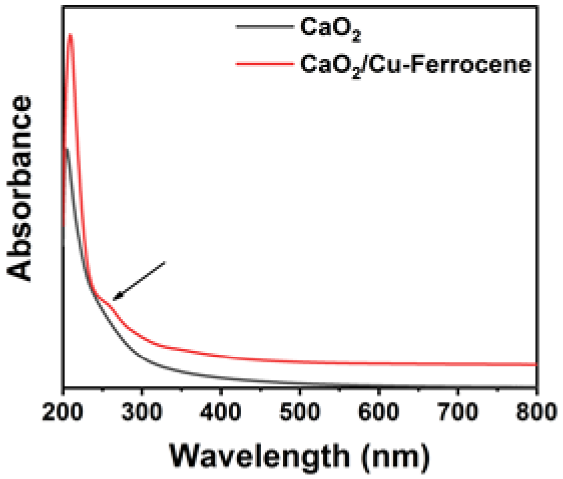

Figure S2. UV-vis spectra of CaO_2 nanoparticles before and after Cu-ferrocene functionalization.

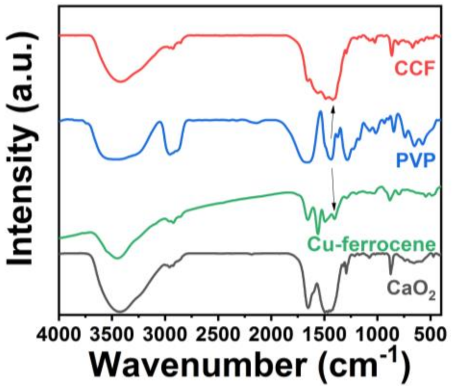

Figure S3. FT-IR spectra of CaO_2/Cu-ferrocene, PVP, Cu-ferrocene and CaO_2.

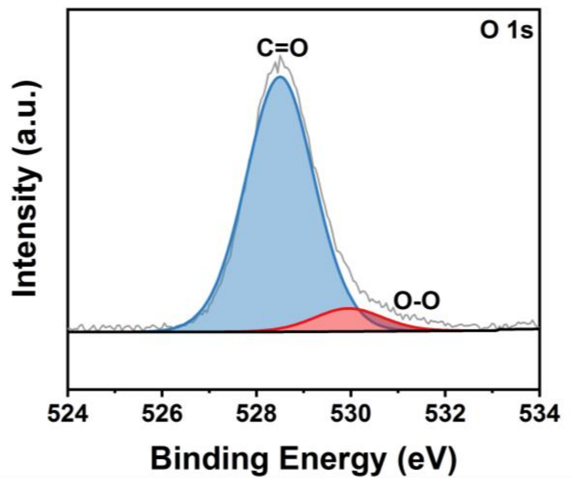

Figure S4. XPS spectrum of O 1s with high magnification.

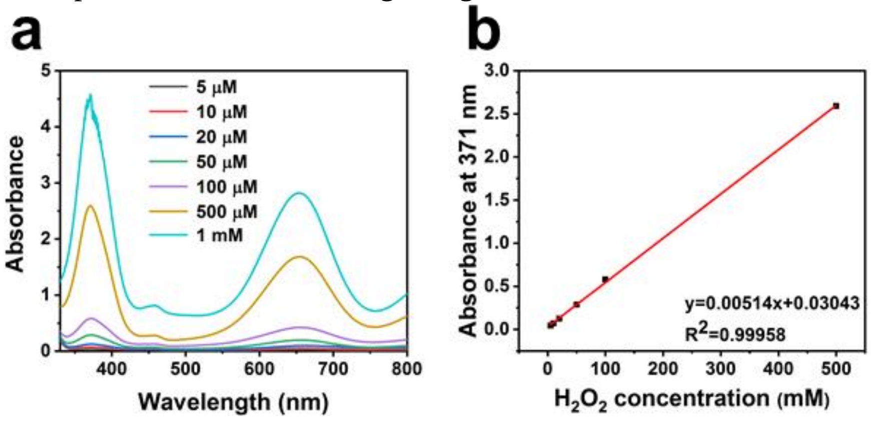

Figure S5. Standard curves of H_2O_2 at the peak of 371 nm by TMB assay: (a) UV-vis absorbance spectra and (b) plotting curve of TMB solution with the addition of different concentrations of H_2O_2.

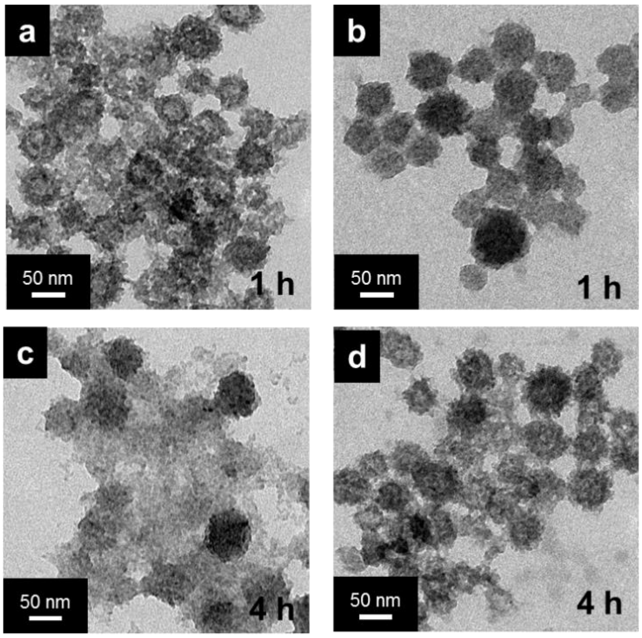

Figure S6. TEM images of (a) CaO_2 and (b) CCF after incubation for 1 h in PBS; (c) CaO_2 and (d) CCF after incubation for 4 h in PBS

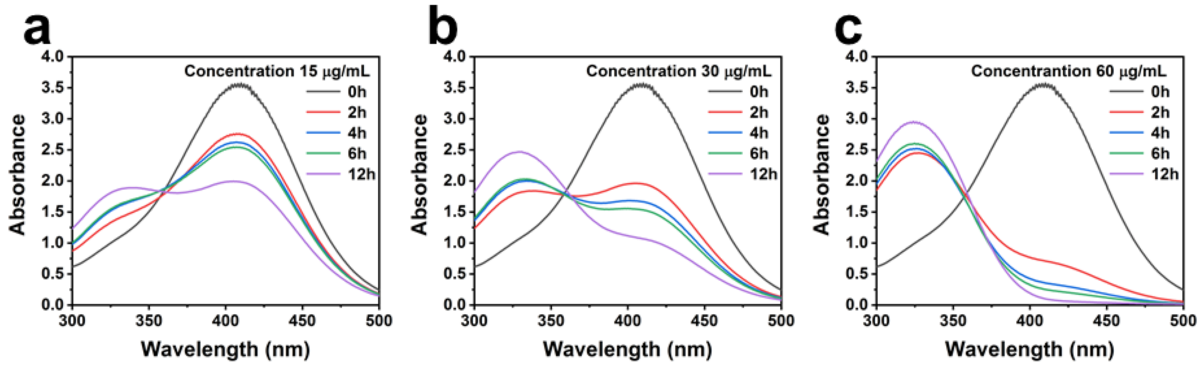

Figure S7. GSH consumption by CaO_2/Cu-ferrocene with concentrations of (a) 15 μg/mL, (b) 30 μg/mL, (c) 60 μg/mL.

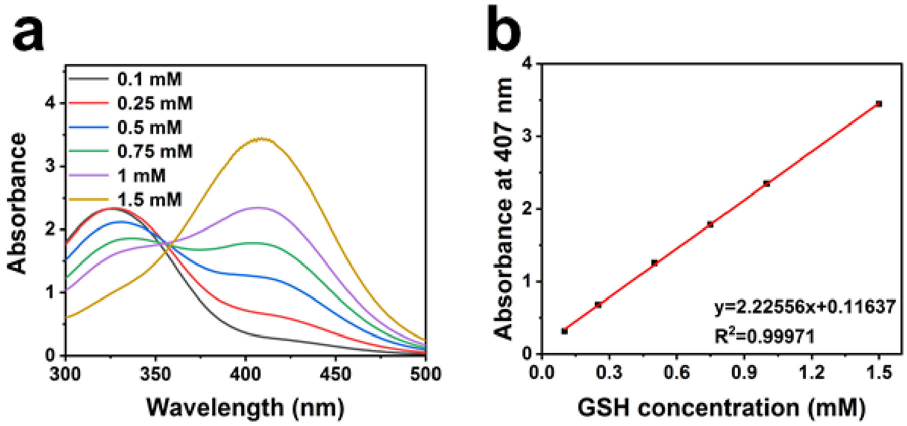

Figure S8. Standard curves of GSH at the peak of 407 nm by DTNB method: (a) UV-vis absorbance spectra and (b) plotting curve of DTNB solution with the addition of different concentrations of GSH.

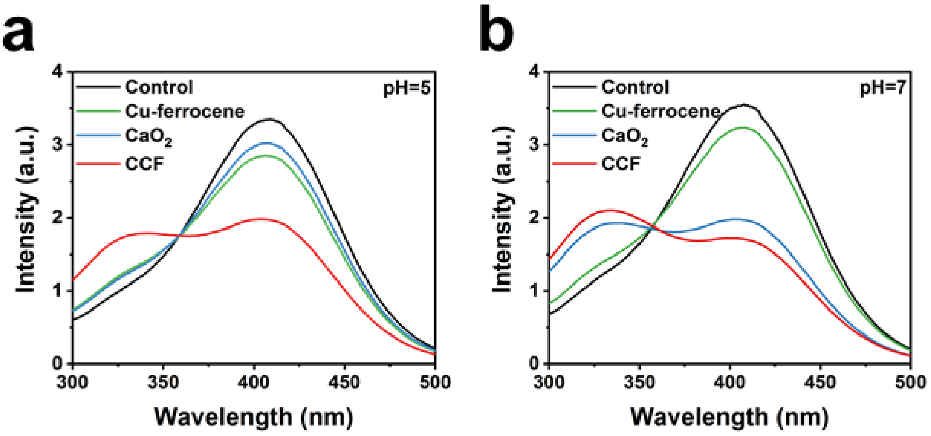

Figure S9. UV-vis absorbance spectra of GSH solutions after the addition of Cu-ferrocene, CaO_2, and CCF, respectively, for 1 h at the pH of (a) 5 and (b) 7.

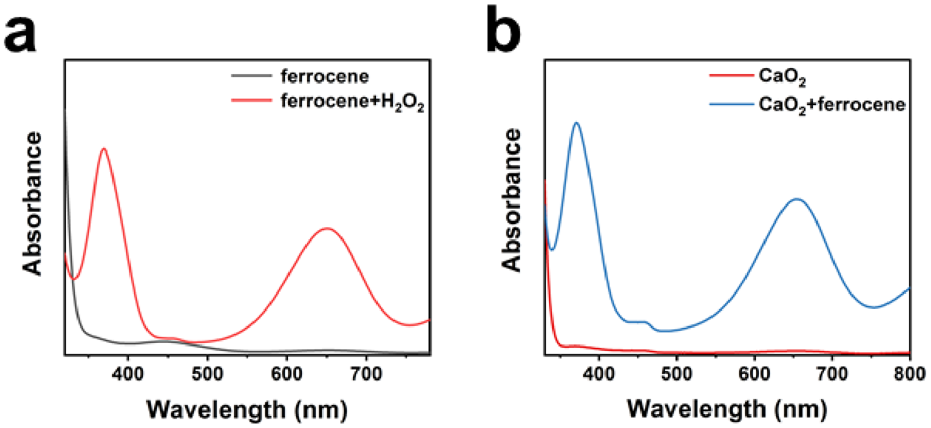

Figure S10. UV-vis absorbance spectra of TMB solution (a) with the addition of ferrocene and ferrocene+H_2O_2; (b) with the addition of CaO_2 and CaO_2+ferrocene (initial pH=5).

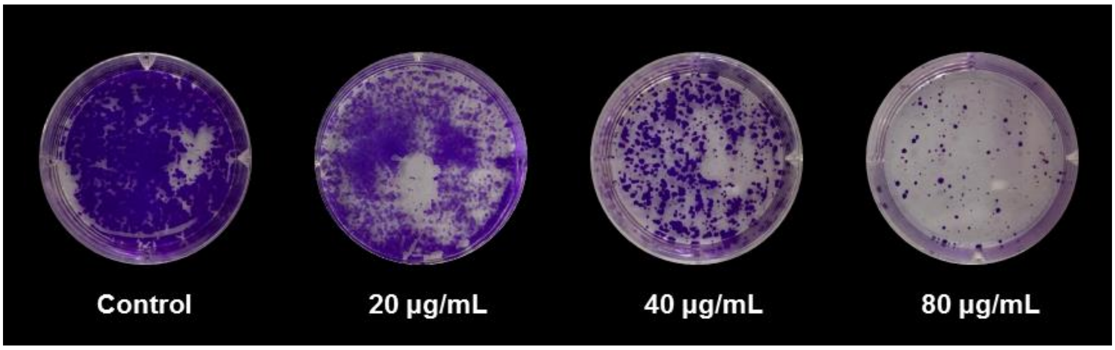

Figure S11. Colony formation of 4T1 cells incubated with different concentrations of CaO_2/Cu-ferrocene nanoparticles.

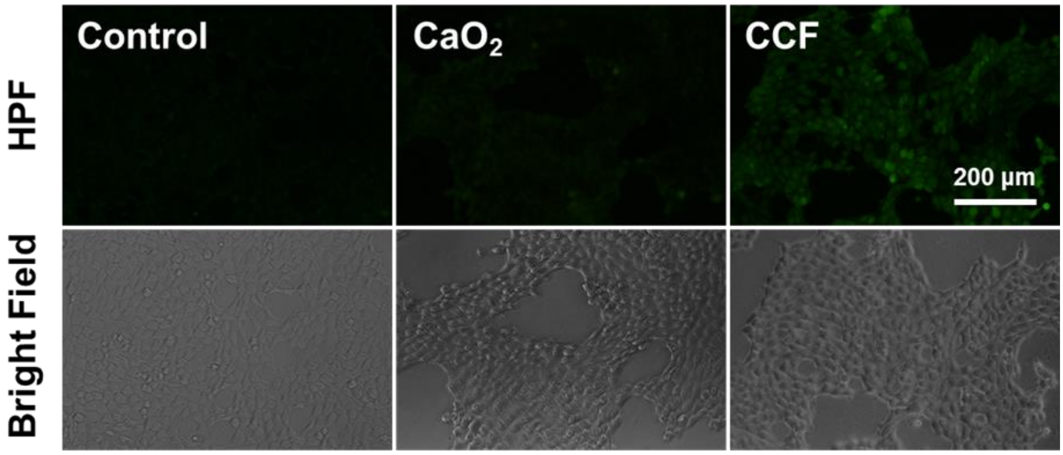

Figure S12. Fluorescence image of HPF stained 4T1 cells after being cultured with CaO_2 and CaO_2/Cu-ferrocene (CCF) for the intracellular •OH detection.

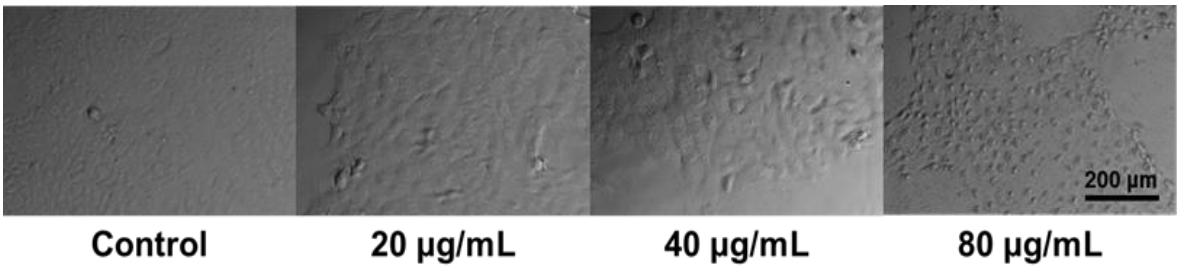

Figure S13. Bright field images of 4T1 cells incubated with different concentrations of CaO_2/Cu-ferrocene after NDA staining for GSH detection.

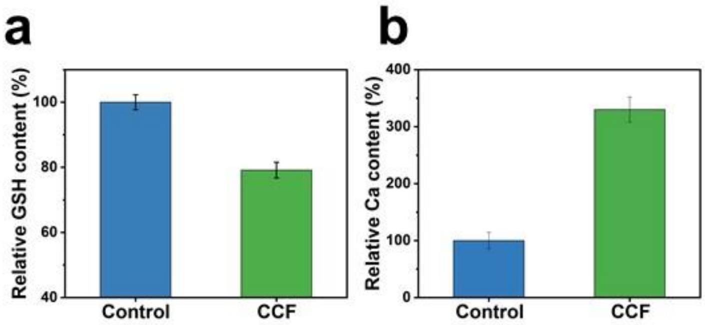

Figure S14. Intracellular (a) GSH and (b) calcium content in 4T1 cells treated without or with CaO_2/Cu-ferrocene.

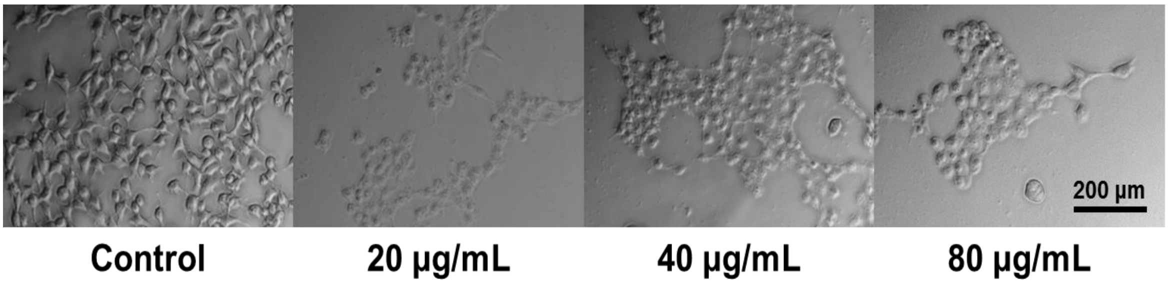

Figure S15. Bright field images of 4T1 cells incubated with different concentrations of CaO_2/Cu-ferrocene after Fluo-4 AM staining for intercellular Ca^{2+} accumulation.
s

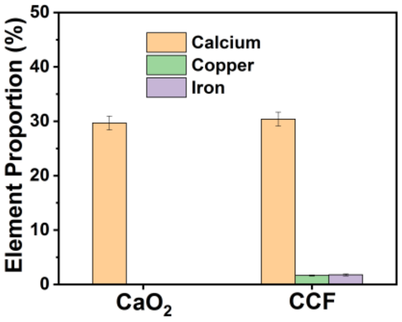

Figure S16. The proportion of each element in CaO_2 and CCF.

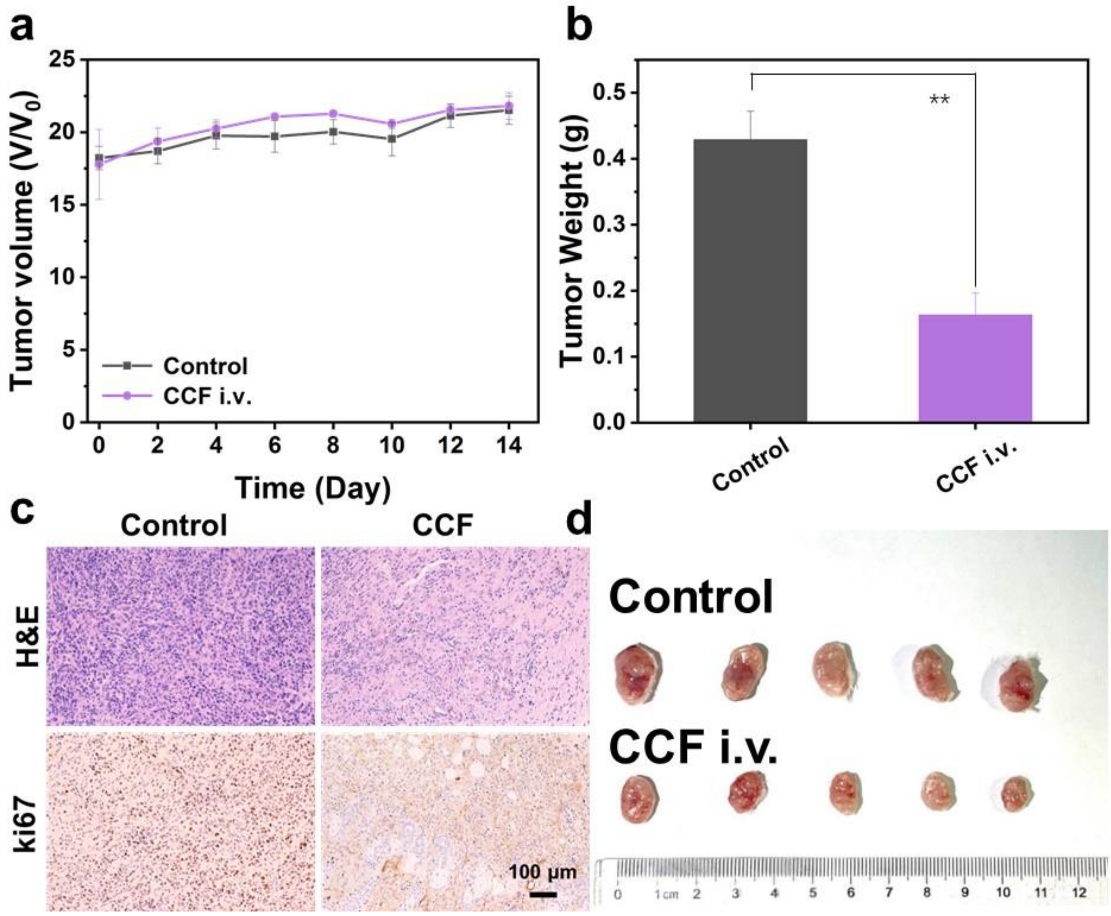

Figure S17. (a) Variation of body weight over 14 days; (b) Tumor weight, (c) images of H&E and ki67 stained tumor slices and (d) tumor photographs from different groups after intravenous injection for 14 days. (***p < 0.001, **p < 0.01, or *p < 0.05.)

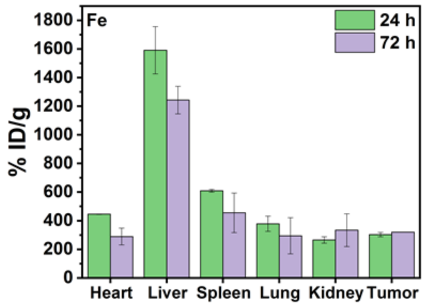

Figure S18. Distribution of Fe in different organs at 24 h and 72 h after the intravenous injection of CCF.

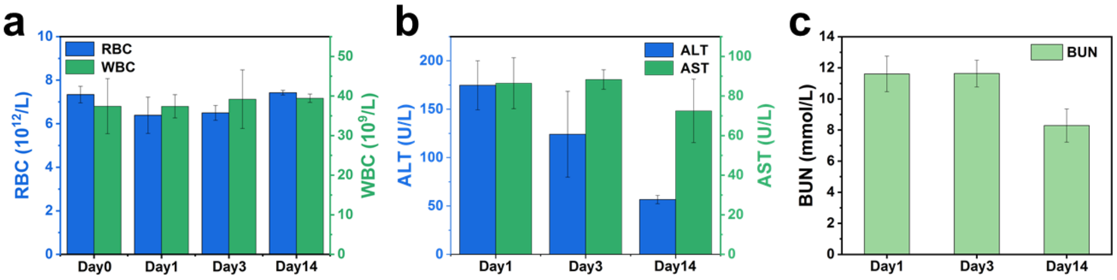

Figure S19. (a) The number of red blood cells (RBC) and white blood cells (WBC); (b) Serum ALT, AST and (c) BUN contents in CCF treated 4T1 tumor-bearing mice (i.v. injection).

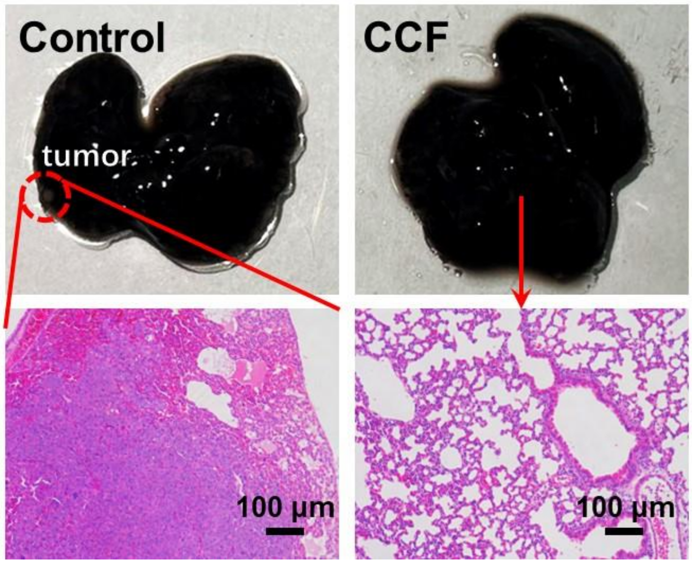

Figure S20. The photographs of the lung in 4T1 tumor-bearing mice after different treatments (red circle represents pulmonary metastasis) and H&E stained slices from lung in control and CCF groups.
